# Infectious Pneumonia

**Published:** 1886-11

**Authors:** 


### Medical News.

INFECTIOUS PNEUMONIA.—In 1881 Pasteur inoculated rabbits with the saliva of a girl who had died of hydrophobia. This produced a blood poisoning in the rabbits which was designated "*salivary septicæmia.*" It was afterward discovered that the saliva of patients suffering from pneumonia was most liable to produce this septicæmia. Banti of Florence has instituted a series of experiments proving that the salivary septicæmia of Pasteur and the disease produced by the inoculation of the pneumonoccus of Fraenkel were identical. He summarizes the results obtained in the Sept. number of *Lo Sperimentale* as follows:

1. The inoculation of the pneumonic excretion constantly produces salivary septicæmia in rabbits.

2. Among the numerous and varied species of bacteria found in the sputa inoculated into rabbits, one only is diffused through the system and becomes pathogenic.

3. The blood of animals dead with salivary septicæmia is capable of reproducing the same affection on inoculation, and its virulence is not reduced by its passage from one animal to another.

This micro-organism is not constantly found in pneumonia. It is most frequently observed in fibrinous pneumonia and in cases where the pleura as well as the lungs are affected.

---

DR. ALMANERA BUTLER, of Chili, South America, advocates what he calls *the water diet* in the treatment of cholera infantum. One of the most prominent symptoms of the disease being intense thirst, it was taken for granted that nature pointed out the required treatment. Pure cold water is given in quantities as large as the child desires. Dr. Luton, of Rheims, was the first to advocate this treatment. The following are the principles advocated by him:

1. Stop all food, for this is the probable cause of the disease.

2. Give pure cold water in unlimited quantities. This acts as a tonic to the intestines and increases the volume of blood.

3. Gradually return to a nutritious diet. Starches and sugar should be prohibited until patient has recovered. This treatment will not interfere with the usual pharmacological remedies. Dr. Luton advocates the hypodermic use of morphine and nitrate of silver internally.

